# Intestinal microbiota in nursery pigs: comments on anatomical location, spatial niches, and approaches to data interpretation

**DOI:** 10.20517/mrr.2026.21

**Published:** 2026-06-12

**Authors:** Alexa R. Gormley, Yesid Garavito-Duarte, Jeonghyeon Son, Sung Woo Kim

**Affiliations:** Department of Animal Science, North Carolina State University, Raleigh, NC 27695, USA.

## INTRODUCTION

In monogastric animals, the intestine is the primary site of food digestion and nutrient absorption, making intestinal function a key determinant of health and growth. Consequently, intestinal health has become a major focus within animal nutrition research^[1,2]^. Increasingly, this work has emphasized the complexity of microbiota-host-immune interactions and the role of the intestinal microbiota in regulating intestinal function^[3,4]^. This commentary aims to highlight key considerations for evaluating the intestinal microbiota in nursery pigs, with particular emphasis on anatomical location, spatial microbial niches, and methodological approaches to data interpretation. Specifically, this commentary discusses the need for a multifaceted approach to understand the role of the intestinal microbiota in nursery pigs.

## SIGNIFICANCE OF THE SMALL INTESTINAL MICROBIOTA

A substantial proportion of studies evaluating the intestinal microbiota in monogastrics rely on fecal samples^[5,6]^, which primarily represent luminal microbial populations of the large intestine. This approach offers practical advantages, including reduced invasiveness and decreased cost. Additionally, because microbial density is greatest in the large intestine, these microbial populations are used to assess microbial diversity and function^[7,8]^. However, in nursery pigs, the relevance of the large intestinal microbiota is less than that of the small intestinal microbiota. Young animals consume highly digestible diets, which reduces the amount of substrates reaching the large intestine. Furthermore, although the microbiota of the large intestine is important for long-term health, its relevance in production systems that prioritize short-term growth performance is low^[9]^. 

In contrast, the small intestine is the primary site of nutrient digestion and absorption^[9]^, directly influencing feed efficiency and growth. The small intestinal microbiota plays a critical role in the development of intestinal structure, digestive function, and immune maturation^[10]^. At weaning, pigs undergo a rapid transition from a milk-based diet to solid feed, which drives significant shifts in microbial populations^[11]^. This period of instability can allow the expansion of opportunistic or pathogenic bacterial populations that are otherwise suppressed by a stable commensal community. Beyond its role in digestion, the small intestine contains a large proportion of the body’s immune cells and serves as a point of communication between the external environment and the host immune system^[12,13]^. Interactions with the microbiota can be beneficial, such as promoting immune tolerance and anti-inflammatory signaling, or harmful, such as activating cascades of inflammatory signaling that damage intestinal tissue. These immune responses can also repartition nutrients away from growth and toward maintenance of immune function, thereby reducing production efficiency. 

It has been well documented that the microbial composition varies along the length of the gastrointestinal tract in pigs^[14-16]^. Changes in substrate availability and environmental conditions result in distinct differences in the microbial communities of the small and large intestine. Therefore, microbial measurements obtained from these regions should be evaluated independently, and in the context of understanding intestinal health in newly weaned pigs, it would be advantageous to examine the microbiota of the small intestine.

## FUNCTIONAL DIFFERENCES BETWEEN LUMINAL AND MUCOSAL MICROBIOTA

Within the intestine, the intestinal microbiota can broadly be categorized as being luminal or mucosal^[17]^. The luminal microbiota is associated with digesta and is primarily involved in nutrient metabolism and fermentation. In contrast, the mucosal microbiota exists in close proximity to the intestinal epithelium and directly interacts with host enterocytes and immune cells. These spatial differences are accompanied by distinct environmental conditions. The mucosal layer provides a stable environment, with the support of mucin as a nutrient source and physical barrier, whereas the luminal environment is more dynamic and is heavily influenced by changes in substrate availability^[18]^. Oxygen gradients further differentiate these communities, leading to distinct differences in the microbial populations within the luminal and mucosal microbiota^[19]^. Given their proximity to the intestinal epithelium, the mucosal microbiota has a greater influence on host immune responses. As such, their evaluation is particularly important when investigating mechanisms related to intestinal health and immune function.

## ABSOLUTE AND RELATIVE ABUNDANCE: INTERPRETIVE CONSIDERATIONS

In addition to spatial considerations, methodological approaches to microbial data analysis play an important role in its interpretation. Relative abundance is commonly used to describe microbial composition^[3-5,14-16]^, however, it is inherently compositional and should be interpreted with caution. Changes in the relative abundance of one taxon may reflect shifts in another taxa, rather than true changes in population size^[20]^. In contrast, absolute abundance provides a direct measure of microbial quantity and is not influenced by the proportional distribution of other taxa^[21,22]^. This approach is particularly valuable when identifying biologically meaningful changes in specific populations or when outliers may distort relative interpretation. Furthermore, absolute abundance facilitates more reliable comparisons across studies, whereas relative abundance data are often difficult to compare due to differences in overall community structure. For these reasons, incorporating absolute abundance alongside relative abundance interpretations can strengthen the biological significance of microbiome data.

## CONCLUSION

The evaluation of the intestinal microbiota in nursery pigs can help in unraveling the relationships among the diet, the intestinal microbiota, and the host. Although a practical approach, reliance on fecal samples as a proxy for the entire gastrointestinal tract overlooks the functional and compositional differences that occur along the length of the gastrointestinal tract, particularly in the small intestine, where nutrient digestion, absorption, and immune interactions are critical to animal performance. Similarly, failing to distinguish between luminal and mucosal microbial populations limits accurate interpretation of direct microbiota-host interactions. Methodological limitations, notably the exclusive use of relative abundance data, can obscure meaningful biological changes and complicate comparisons across studies. Incorporating both the absolute and relative abundance measures provides a more complete view of compositional dynamics within the microbiota. Collectively these considerations highlight the need for more intentional and targeted approaches to evaluating the intestinal microbiota in the context of nursery pig nutrition.

## DECLARATIONS

### Copyright

© The Author(s) 2026.
